# World Health Organization Guideline Development: An Evaluation

**DOI:** 10.1371/journal.pone.0063715

**Published:** 2013-05-31

**Authors:** David Sinclair, Rachel Isba, Tamara Kredo, Babalwa Zani, Helen Smith, Paul Garner

**Affiliations:** 1 Department of Clinical Sciences, Liverpool School of Tropical Medicine, Liverpool, United Kingdom; 2 South African Cochrane Centre, Cape Town, South Africa; 3 Department of International Public Health, Liverpool School of Tropical Medicine, Liverpool, United Kingdom; University of Washington, United States of America

## Abstract

**Background:**

Research in 2007 showed that World Health Organization (WHO) recommendations were largely based on expert opinion, rarely used systematic evidence-based methods, and did not follow the organization's own “Guidelines for Guidelines”. In response, the WHO established a “Guidelines Review Committee” (GRC) to implement and oversee internationally recognized standards. We examined the impact of these changes on WHO guideline documents and explored senior staff's perceptions of the new procedures.

**Methods and Findings:**

We used the AGREE II guideline appraisal tool to appraise ten GRC-approved guidelines from nine WHO departments, and ten pre-GRC guidelines matched by department and topic. We interviewed 20 senior staff across 16 departments and analyzed the transcripts using the framework approach. Average AGREE II scores for GRC-approved guidelines were higher across all six AGREE domains compared with pre-GRC guidelines. The biggest changes were noted for “Rigour of Development” (up 37.6%, from 30.7% to 68.3%) and “Editorial Independence” (up 52.7%, from 20.9% to 73.6%). Four main themes emerged from the interviews: (1) high standards were widely recognized as essential for WHO credibility, particularly with regard to conflicts of interest; (2) views were mixed on whether WHO needed a single quality assurance mechanism, with some departments purposefully bypassing the procedures; (3) staff expressed some uncertainties in applying the GRADE approach, with departmental staff concentrating on technicalities while the GRC remained concerned the underlying principles were not fully institutionalized; (4) the capacity to implement the new standards varied widely, with many departments looking to an overstretched GRC for technical support.

**Conclusions:**

Since 2007, WHO guideline development methods have become more systematic and transparent. However, some departments are bypassing the procedures, and as yet neither the GRC, nor the quality assurance standards they have set, are fully embedded within the organization.

## Introduction

The World Health Organization (WHO) is a leading producer of healthcare recommendations, guiding and informing policy worldwide, particularly for low and middle income countries. Research published in 2007 however demonstrated that WHO recommendations were based mainly on expert opinion and rarely used systematic evidence-based methods [1]. Internal ‘Guidelines for Guidelines’ were in place since 2003, but the organization lacked an effective mechanism to enforce the expected standards [2]–[4].

This public criticism prompted the WHO to establish a ‘Guidelines Review Committee’ (GRC), composed of both internal staff and external advisors, tasked with implementing and overseeing quality assurance [5]. The GRC re-established a set of guideline development standards and adopted the GRADE (Grading of Recommendations Assessment, Development and Evaluation) approach to formulating evidence-based recommendations [6]. Supported by a small secretariat, the GRC now expects to review new and updated guideline proposals at the planning stage and again before publication [7]. In this paper we evaluate WHO guideline documents against international standards pre and post formation of the GRC, and explore senior staff's perceptions of the GRC and the new procedures.

## Methods

### Ethics statement

The study protocol was discussed with both the Liverpool School of Tropical Medicine Ethics Committee and the WHO Ethics Review Committee and received a formal written waiver. Permission to conduct the study was granted by the Assistant Director General of the WHO. All participants in the study provided written informed consent prior to being interviewed.

### Guideline appraisal

We sampled WHO guidelines, pre- and post-GRC, from a spread of WHO departments and across a range of broad topic areas (prevention, diagnosis, treatment, and health systems), using a ‘matched’ before-and-after study design.

To fully evaluate the impact of the changes we chose guidelines published online during 2010 as the initial post-GRC sampling frame (most guidelines published in 2008/09 only partially implemented the changes as they were already in progress prior to the establishment of the GRC). We randomly selected one guideline from each department that published during 2010, and matched this with a pre-GRC guideline from the same department and broad topic area. Where possible this match was with an earlier edition of the same document.

We used the AGREE II appraisal tool to assess the methods and presentation of each guideline using 23 criteria across six domains: scope and purpose, stakeholder involvement, rigour of development, clarity of presentation, applicability, and editorial independence [8]. AGREE II provides extensive explanatory notes for each criterion to guide the appraisal and improve consistency across reviewers [9].

All four assessors (DS, RI, TK and BZ) completed the AGREE II online training modules prior to beginning the study, and to improve standardization, one guideline was appraised and discussed extensively by all four. Each guideline was allocated randomly to two assessors who worked independently but were not blinded to the year of publication. Each assessor initially allocated a score between 1 and 7 for each criterion, and scores were then aggregated across assessors and converted to a percentage for each domain. Wide disagreement in scores was resolved by a third assessor.

DS and PG, who have participated in the development of guidelines in malaria, were excluded from appraising guidelines from the Global Malaria Programme.

### WHO staff interviews

We interviewed senior staff from 18 WHO departments, representing departments experienced with publishing GRC-approved guidelines, others who had not yet been through the process, the GRC chair and members of the secretariat. The GRC secretariat assisted in this selection process to ensure a spread of ‘supporters and opponents’. Following written informed consent, each person was interviewed by two researchers (PG and DS) in April 2012 using a topic guide (Appendix S1). Interviews were audio-recorded and fully transcribed. We used framework analysis to develop a thematic framework which we used to code and organise the data and, through an iterative process with regular discussion, we assembled related codes to form the main themes [10]. The initial draft manuscript was distributed to all participants for comment and correction of factual error or interpretation.

## Results

### Guideline appraisal

Seventeen GRC-approved guidelines were published online in 2010, from nine different WHO departments spread across six departmental clusters. Of note, four were published by the HIV department and five by STOP TB. From these seventeen, ten were selected for formal appraisal: one from each of the nine departments and one additional guideline from the HIV department as it was the only guideline addressing diagnostic questions. Five guidelines addressed questions about treatment, two about safety or illness prevention, and two provided health system guidance.

The ten matched guidelines were published between 2003 and 2008 by the same nine WHO departments, all pre-dating the GRC approval process. Of these, five were earlier editions of the same guideline and five were earlier guidelines addressing a similar topic area.

Mean scores for all six domains of the AGREE II appraisal were higher in the ten GRC-approved guidelines than in the ten older ‘matched’ guidelines (Table 1; full assessments in Appendix S2). Seven of the matched pairs showed substantial improvement against almost all the criteria, while three made little improvement or declined.

**Table 1:** AGREE II scores for guidelines published pre and post formation of the GRC.^1^

	Mean scores^3^ (%)				
	Pre GRC^2^	Post GRC^2^			
AGREE II domain	(n = 10)	(n = 10)	Mean difference (%)	Median change in domain score (%)	Range
**Scope and Purpose** 4	62.2	80.4	+18.2	+12.5	−3 to +39
**Stakeholder Involvement** 5	49.8	61.2	+11.4	+18.0	−33 to +47
**Rigour of Development** 6	30.7	68.3	+37.6	+53.5	−26 to +76
**Clarity of Presentation** 7	60.9	78.2	+17.3	+23	−47 to +52
**Applicability** 8	49.1	61.6	+12.5	+16.5	−22 to +65
**Editorial Independence** 9	20.9	73.6	+52.7	+67	−21 to +92

1 Three additional guidelines were appraised, published since 2010 but known to have bypassed or not used the GRC approval process. These documents scored at levels similar to the pre-GRC guidelines and can be seen in Appendix S3.

2 Each guideline was appraised by at least two assessors working independently and the individual scores aggregated.

3 Mean scores were calculated across all ten guidelines for each domain.

4 Scope and purpose concerns the overall aim of the guideline, the scope of the questions, and the target audience.

5 Stakeholder involvement looks at the extent to which the guideline development process included the views of all appropriate stakeholders, including the intended users of the guideline and those affected by the recommendations.

6 Rigour of development examines the process used to search for, synthesize, and appraise evidence, formulate recommendations, and keep them updated.

7 Clarity of presentation concerns the general language, structure, and format of the guideline.

8 Applicability requires adequate consideration of the likely barriers and facilitators to implementation, including resource considerations, and advice or tools to improve uptake and implementation.

9 Editorial independence concerns the adequate declaration and management of potential conflicts of interest related to the funding body or the guideline group members.

‘Rigour of Development’ and ‘Editorial Independence’ were the lowest scoring domains across the pre-GRC guidelines with eight out of ten guidelines scoring less than 40% for both domains. Substantial improvements were seen in seven of the GRC-approved guidelines, with eight of the ten guidelines now scoring greater than 60% for both domains. ‘Stakeholder Involvement’ and ‘Applicability’ were now the lowest scoring domains in GRC-approved guidelines.

During the research we identified two additional documents which had bypassed the GRC process, and were informed that one guideline group had a formal ‘waiver’ on the GRC process. An AGREE II appraisal of these three documents gave scores similar to pre-GRC guidelines (Appendix S3).

### Interviews with senior staff

The 20 interviews were conducted with ten heads of departments (Directors), seven senior technical staff who had been involved with guideline development (Co-ordinators, Medical Officers and Technical Officers), and three others directly involved with the GRC. Through careful analysis of the content of these interviews we identified four main themes (Table 2).

**Table 2:** Four key emergent themes.

1. High standards essential for credibility
The normative role of the WHO was widely recognised as a core function and guidelines as one of the most visible products. High standards were considered essential for defending the WHO against criticism, particularly with regard to conflicts of interest.

2. Mixed views on the need for a single quality assurance process
While the senior directors universally advocated for high standards, opinions were mixed on the need for a single quality assurance process across the organization. Some questioned the power given to the current GRC and some purposefully avoided the process, while others now viewed it as a valuable resource and an essential mechanism for the organization.

3. Uncertainties about applying the GRADE approach
Several uncertainties relating to the GRADE approach were noted. While departmental staff tended to concentrate on discussing the technicalities and bureaucracy of GRADE, those from the GRC emphasized the general principles underlying a transparent, systematic, and explicit process, and remained concerned that even these were not yet fully institutionalized.

4. Technical capacity to implement the new standards is variable
Many who had been through the GRC process described it as a steep learning curve, both for them and for the external experts who were often equally unfamiliar with these methods. It was clear that many departments were looking to the GRC to provide technical support alongside quality assurance and the GRC themselves expressed the lack of both financial and personnel capacity to do this.

#### 1) High standards are essential for WHO credibility (Table S1)

Most directors acknowledged that the criticism levelled at the organization in 2007 was fair and that many WHO guidelines and recommendations published prior to this were of low quality.

Some noted that these deficiencies were not universal and gave examples of evidence-based systematic processes in use well before the GRC. These interviewees viewed the recent changes in process as relatively minor for them but essential for raising standards across the organization.

Senior staff widely recognized the normative role of the WHO as central to its mission, and guidelines as one of the most visible products on which the organization is judged. High standards were noted as important by interviewees to defend the WHO against criticism (particularly with regard to conflicts of interest); to improve the reliability of recommendations; and to ensure the future credibility and position of the organization.

#### 2) Mixed views on the need for a single quality assurance mechanism (Table S2)

While the interviewees universally advocated high standards, opinions were mixed on the need for a single quality assurance mechanism across the organization, with some questioning the power given to the current GRC.

One director expressed strong opinions against the GRC and against a perceived loss of autonomy, describing the process as a “monstrous bureaucracy”; citing negative early experiences with the GRC, and scepticism from external members of guideline panels. Another director questioned whether the GRC should have the power to block documents at either the planning or publication stage, and whether the GRC had the technical capacity to make such a judgement.

Others were very positive about the GRC, its influence, and how it had helped them. Some admitted to similar initial concerns – of fear about the ‘guideline police’ delaying and unnecessarily complicating the process – but through positive experiences now viewed the GRC as a valuable resource and essential as one of the few quality assurance mechanisms within the WHO.

Representatives of the GRC reported constructive partnerships with many departments but raised examples of groups bypassing the GRC process, either by “game-playing” (calling the document a policy brief or meeting report instead of a guideline) or wilfully through disregard for the GRC and the procedures.

#### 3) Uncertainties about applying the GRADE approach (Table S3)

Several areas of uncertainty relating to the GRADE approach were noted. While departmental staff tended to concentrate on discussing the technicalities and bureaucracy of the process, those from the GRC emphasised the general principles underlying a transparent, systematic, and explicit process, and remained concerned that even these were not yet fully institutionalized.

Most interviewees agreed that the GRADE approach to assessing quality of evidence worked well for clinical questions and recommendations, but some were uncertain about whether these methods could be used for health systems or implementation guidance. However, we did hear one example of a group using GRADE for a health systems guideline with enthusiasm. This group felt the additional efforts had increased the clarity and usefulness of their recommendations.

Some interviewees discussed uncertainties about how to apply the GRADE approach when the evidence was of very low quality or when the recommendation seemed obviously ‘common sense’. In these instances, some felt forced to search for evidence when it was considered unhelpful or necessarily time-consuming. Some also expressed concerns that if applied wrongly, a formulaic approach to GRADE could mislead or detract people from addressing the right questions with the most appropriate methods, and lead to poorly thought-out documents, or ill-advised recommendations.

Many of the interviewees acknowledged that making recommendations that were intended to inform policies in many different settings was not straightforward: disease burden, health infrastructure and financing, and cultural values and preferences are all different and need to be taken into account. There was uncertainty about how to incorporate these considerations into WHO documents or how to provide the necessary contextual guidance within the framework of existing methods.

#### 4) Variable capacity to implement the new standards (Table S4)

Many who had been through the GRC approval process described it as a steep learning curve, both for them and for the external experts who were often equally unfamiliar with these methods. Several directors and technical staff had attended educational sessions conducted by the GRC and valued these highly.

Some senior staff stated that the technical expertise to apply these new methods was not widespread within the organization and others admitted that their progress so far had been reliant on just one or two highly skilled individuals within the department. Some however, appeared fully engaged in the process, were talking with the GRADE working group, and seemed keen to further improve the methods for application to global guidelines.

Several directors had hired new staff with the specific skills required to implement the procedures and prepare the documents. Some reported that GRC members had provided useful methodological input to the expert meetings and some had brought in external GRADE methodologists to sit on the expert panels. A few directors did not seem to understand the concept of ‘guideline methodology’ and were content to rely on the statistical understanding of existing expert members who were usually academic researchers or content experts.

While some departments seemed to be having difficulty fitting the requirements of the GRC methods around the existing framework of long-established expert groups or committees, others appeared more innovative, readily disbanding such groups and finding new ways to garner expert advice at appropriate stages of the process.

## Discussion

Four years after the GRC was formed, our AGREE II evaluation demonstrates that the transparency of WHO guidelines processes has improved and the organization is making wider use of systematic evidence appraisal. However, we found wide differences between groups in their capacity to implement these changes and in their willingness to participate in the GRC procedures; with some groups embracing it enthusiastically, some bypassing it, and some simply rejecting it.

The AGREE II appraisal has several limitations. Firstly, the AGREE II tool is unable to fully assess the ‘appropriateness’ of the final recommendations or ‘use-ability’ of the final document – both highly important considerations. Despite this, reporting of funding sources, conflicts of interests, and guideline methodology are considered minimum standards internationally and are essential to the future credibility of the organization [11]. Secondly, the study is susceptible to bias as the assessors were not blinded to year of publication. This is unlikely to fully explain the findings however, as the changes are both large and involve clearly identifiable items. For example; within the majority of matched pairs there was transition from almost complete absence of a methods section in the pre-GRC guideline to a fairly complete description in the GRC-approved document. Despite the small sample size, this study therefore provides fairly robust evidence of change within some guideline groups. Thirdly, the uncontrolled nature of the study is unable to prove that the observed changes are a result of the GRC. Other factors may also be important, such as an increased involvement of guideline methodologists on WHO panels, a change in attitudes of WHO department directors as a result of the external criticism, and simply a gradual improvement over time. However, from the interviews with staff members it is clear that many within the organization consider the GRC secretariat and committee to be major players in overseeing, facilitating, and enforcing this change.

From our own experience and understanding of the GRADE approach, the concerns raised during the interviews seem to relate to poor understanding of the process and a lack of embedded institutional capacity, rather than deficiencies in the methods themselves. Effective use of the GRADE approach requires high quality, well-constructed systematic reviews, followed by critical and thoughtful analysis and interpretation of the data by the guideline panels, both of which can prolong and complicate the guideline development process without adequate planning and in-house technical capacity. Difficulties such as ‘common-sense’ recommendations are common across guideline developers and have been addressed elsewhere by the GRADE Working Group [12]. On the other hand, developing global guidelines for widely differing settings and systems entail challenges that are less common. Many guideline groups have brought in external guideline specialists, who are perhaps uniquely placed to offer insights into the difficulties encountered with using GRADE and to suggest appropriate solutions to continuing this improvement process. Their opinions would be extremely valuable in providing insight into how the organization is learning and how to further institutionalise this process. Indeed, the WHO could take a leadership role in collaboration with their methodological partners in advancing appropriate methods in guideline development.

At present, the GRC itself appears fragile and the procedures it has put in place could yet be derailed by departments wanting to do things their own way. Indeed, some interviewees recounted the near collapse of the GRC in late 2010/11 through mismanagement, and it is a credit to the organization that it has survived.

## Conclusions

WHO procedures for developing guidelines have improved considerably since the GRC was established, with wider use of systematic methods, improved transparency and better management of potential conflicts of interest. The interviews with senior WHO staff support the conclusion that a large part of these improvements can be attributed to the GRC.

However, as yet neither the GRC, nor the changes implemented by them, are fully embedded within the organization. Political support and greater resourcing are required to institutionalize the principles and procedures, and move forward with further improvements.

## Supporting Information

Appendix S1 **Draft Interview Guide.**
(DOCX)

Appendix S2 **Full AGREE II appraisal scores for pre- and post-GRC guidelines.**
(DOCX)

Appendix S3 **AGREE II appraisal scores for three recent guidelines that did not seek GRC approval.**
(DOCX)

Table S1 **Theme 1: High standards essential for credibility.**
(DOCX)

Table S2 **Theme 2: Mixed views on the need for a single quality assurance process.**
(DOCX)

Table S3 **Theme 3: Uncertainties about applying the GRADE methods.**
(DOCX)

Table S4 **Theme 4: Variable capacity to implement the new standard.**
(DOCX)
