# Correction: World Health Organization Guideline Development: An Evaluation

**DOI:** 10.1371/annotation/fd04e7c6-0d40-4d2c-a382-c5ad10074c99

**Published:** 2013-09-13

**Authors:** David Sinclair, Rachel Isba, Tamara Kredo, Babalwa Zani, Helen Smith, Paul Garner

The affiliation of the third and fourth authors is incorrect. The correct affiliation is: 2 South African Cochrane Centre, South African Medical Research Council, Cape Town, South Africa. 

