# Supplementary material for: World Health Organization Guideline Development: An Evaluation
Source: PLoS One. 2013 May 31;8(5):e63715. doi: 10.1371/journal.pone.0063715 (PMC3669321; doi:10.1371/journal.pone.0063715)
Supplement: Appendix S1 — Draft Interview Guide. (DOCX) [file pone.0063715.s001.docx]

**Draft Interview Guide**

***Background***

1. Can you give us some examples of how you have been involved with guideline development?

***The GRC process***

2. Do you have any views on the situation with WHO guideline development prior to the formation of the GRC?

3. Do you think the GRC has had any influence on the way things are done?

Have things improved/got worse

4. Have you any experience of using or interpreting GRADE?

What do you think about GRADE?

***Agree criteria***

5. What do you think about lay involvement and consumer representation on guideline panels?

Have you any examples of where it has worked/not worked?

6. Are the guidelines that you have been involved in are as clear/short/ well presented as you would like?

Are there any things you would like to see changed?

7. WHO guidelines are often made on a global basis. How well do you think regional, national or sub-national variations are addressed?

How do you think national policy makers should use WHO global guidelines?

Do they need to reappraise the evidence for themselves? Or should they simply follow the WHO advice?

8. How well do you think WHO guideline groups avoid conflicts of interest or industry pressures?

Can you think of ways it could be done better?

9. What do you think of the way Guideline panels are currently selected?

Could the process be improved? What are your suggestions?
