# Supplementary material for: World Health Organization Guideline Development: An Evaluation
Source: PLoS One. 2013 May 31;8(5):e63715. doi: 10.1371/journal.pone.0063715 (PMC3669321; doi:10.1371/journal.pone.0063715)
Supplement: Appendix S2 — Full AGREE II appraisal scores for pre- and post-GRC guidelines. (DOCX) [file pone.0063715.s002.docx]

| WHO  Dept. | Broad  guideline area | Mean scores for the six AGREE II domains (%) | | | | | | | | | | | | | | | | | |
| --- | --- | --- | --- | --- | --- | --- | --- | --- | --- | --- | --- | --- | --- | --- | --- | --- | --- | --- | --- |
|  |  | Scope and Purpose | | | Stakeholder Involvement | | | Rigour of Development | | | Clarity of Presentation | | | Applicability | | | Editorial Independence | | |
|  |  | Pre-GRC | Post-GRC | Change | Pre-GRC | Post-GRC | Change | Pre-GRC | Post-GRC | Change | Pre-GRC | Post-GRC | Change | Pre-GRC | Post-GRC | Change | Pre-GRC | Post-GRC | Change |
| MCH | Treatment | 64 | 78 | +14 | 42 | 83 | +41 | 13 | 89 | +76 | 56 | 83 | +27 | 71 | 65 | -6 | 0 | 92 | +92 |
| GMP | Treatment | 83 | 94 | +11 | 72 | 67 | -5 | 80 | 92 | +12 | 86 | 97 | +11 | 46 | 69 | +23 | 83 | 100 | +17 |
| HIV-A | Treatment | 72 | 100 | +28 | 64 | 92 | +28 | 28 | 95 | +67 | 81 | 100 | +19 | 27 | 77 | +50 | 0 | 92 | +92 |
| HIV-B | Diagnosis | 50 | 89 | +39 | 36 | 58 | +22 | 10 | 84 | +74 | 42 | 94 | +52 | 48 | 69 | +21 | 0 | 63 | +63 |
| STB | Treatment | 75 | 86 | +11 | 31 | 78 | +47 | 16 | 70 | +54 | 58 | 86 | +28 | 63 | 79 | +16 | 21 | 88 | +67 |
| EHT | Prevention | 61 | 64 | +3 | 64 | 50 | -14 | 34 | 33 | -1 | 100 | 53 | -47 | 79 | 40 | -39 | 0 | 67 | +67 |
| HRH | Health systems | 64 | 97 | +33 | 50 | 64 | +14 | 18 | 93 | +75 | 58 | 97 | +39 | 73 | 90 | +17 | 17 | 96 | +79 |
| GIP | Treatment | 75 | 82 | +7 | 78 | 53 | -25 | 90 | 64 | -26 | 89 | 88 | -1 | 67 | 45 | -22 | 67 | 63 | -4 |
| CHP | Prevention | 39 | 78 | +39 | 25 | 64 | +39 | 10 | 63 | +53 | 36 | 67 | +31 | 2 | 67 | +65 | 0 | 75 | +75 |
| VIP | Health systems | 39 | 36 | -3 | 36 | 3 | -33 | 8 | 0 | -8 | 3 | 17 | +14 | 15 | 15 | 0 | 21 | 0 | -21 |
| Mean across departments: | | 62.2 | 80.4 | +18.2 | 49.8 | 61.2 | +11.4 | 30.7 | 68.3 | +37.6 | 60.9 | 78.2 | +17.3 | 49.1 | 61.6 | +12.5 | 20.9 | 73.6 | +52.7 |

**The departments and guidelines assessed were:**

Department of Maternal, Newborn, Child and Adolescent Health (MCH): HIV and Infant Feeding (2003), and Guidelines for Infant Feeding in the Context of HIV (2010)

Global Malaria Programme (GMP): Malaria Treatment Guidelines; First Edition (2006) and Malaria Treatment Guidelines; Second Edition (2010)

HIV/AIDS Department (HIV): Antiretroviral Therapy for Adults and Adolescents (2006), and Antiretroviral Therapy for HIV Infection in Adults and Adolescents (2010)

HIV/AIDS Department (HIV): Provider Initiated HIV Testing and Counselling (2007), and WHO Recommendations on Diagnosis of HIV in Infants and Children (2010)

Stop TB Department (STB): Treatment for Tuberculosis (2003), and Guidelines for the Management of Tuberculosis (2010)

Department of Essential Health Technologies (EHT): WHO Guidelines on Hand Hygiene in Health Care (2006), and Best Practices for Injections and Related Procedures (2010)

Department for Human Resources for Health (HRH): Scaling Up, Saving Lives (2008), and Increasing Access to Health Workers in Remote and Rural Areas through Improved Retention (2010)

Global Influenza Programme (GIP): WHO Rapid Advice on the Pharmacological Management of Influenza (2006), and WHO Guidelines on the Pharmacological Management of Pandemic Influenza (2010)

Chronic Diseases and Health Promotion (CHP): Prevention of Blindness from Diabetes (2006), and Global Recommendations: Physical Activity for Health (2010)

Violence and Injury Prevention and Disability (VIP): Guideline on Essential Trauma Care (2003), and Guidelines on Community-based Rehabilitation (2010)
