# Supplementary material for: World Health Organization Guideline Development: An Evaluation
Source: PLoS One. 2013 May 31;8(5):e63715. doi: 10.1371/journal.pone.0063715 (PMC3669321; doi:10.1371/journal.pone.0063715)
Supplement: Appendix S3 — AGREE II appraisal scores for three recent guidelines that did not seek GRC approval. (DOCX) [file pone.0063715.s003.docx]

| AGREE II domain | AGREE II score  (%) | | | Mean  (%) |
| --- | --- | --- | --- | --- |
|  | Document 1^1^ | Document 2^2^ | Document 3^3^ |  |
| Scope and Purpose | 22 | 89 | 53 | 54.7 |
| Stakeholder Involvement | 31 | 42 | 31 | 34.7 |
| Rigour of Development | 26 | 68 | 50 | 48.0 |
| Clarity of Presentation | 50 | 89 | 50 | 63 |
| Applicability | 23 | 63 | 17 | 34.3 |
| Editorial Independence | 0 | 63 | 71 | 46.7 |

**We evaluated the following documents which had not gone through the GRC approval process:**

^1^ Pneumococcal vaccines WHO position paper 2012 (*Weekly Epidemiological Record*. 2012; 14(87): 129–44), plus all related web appendices including a systematic review and GRADE tables. (The WHO Strategic Advisory Group of Experts (SAGE) on Immunization has a waiver on the GRC process).
^2^ WHO Policy Recommendation: Seasonal Malaria Chemoprevention (SMC) for *Plasmodium falciparum* malaria control in highly seasonal transmission areas of the Sahel sub-region in Africa (<http://www.who.int/malaria/publications/atoz/who_smc_policy_recommendation/en/>), plus all related web appendices
^3^ First global consensus for evidence-based management of the haematopoietic syndrome resulting from exposure to ionizing radiation (*Disaster Med Public Health Preparedness.* 2011;5:202-12)
