# Supplementary material for: World Health Organization Guideline Development: An Evaluation
Source: PLoS One. 2013 May 31;8(5):e63715. doi: 10.1371/journal.pone.0063715 (PMC3669321; doi:10.1371/journal.pone.0063715)
Supplement: Table S1 — Theme 1: High standards essential for credibility. (DOCX) [file pone.0063715.s004.docx]

| Theme 1: High standards essential for credibility |
| --- |
| ‘If we cannot produce, now seriously, cannot produce guidelines of the maximum possible quality and as much as possible evidence based… then the credibility’s at stake, and that’s the issue here.’ *(Director, Interview 7)* |
| ‘Yeah and it’s a sort of a uniform approach to the normative role of WHO which should be a strength of WHO… if everybody follows the same process systematically and so on then it only strengthens the role of the organisation, the position of the organisation.’ *(Technical Officer, Interview 11)* |
| ‘The Guidelines Review Committee has a very important role to play to eliminate all those kind of personal ways of doing things and just some group of experts that could be your friends and they would decide to do something and… you make it a WHO recommendation.’ *(Coordinator, Interview 19)* |
| ‘We didn’t have in the past, for instance, conflict of interest process… when you participate in meetings. We didn’t have this kind of thing. We had to introduce that because a lot of money coming from industry and all that.’ *(Director, Interview 15)* |
| ‘We were following more or less what GRC brought as standards before GRC existed. So in that sense we were ready to work according to that methodology. But for a lot of other departments it completely changed the way they were working and there I think there [have] been more delays or struggles in adapting.’ *(Technical Officer, Interview 20)* |
