# Supplementary material for: World Health Organization Guideline Development: An Evaluation
Source: PLoS One. 2013 May 31;8(5):e63715. doi: 10.1371/journal.pone.0063715 (PMC3669321; doi:10.1371/journal.pone.0063715)
Supplement: Table S2 — Theme 2: Mixed views on the need for a single quality assurance process. (DOCX) [file pone.0063715.s005.docx]

| Theme 2: Mixed views on the need for a single quality assurance process |
| --- |
| ‘Partly they want to be the police of everything... they want to decide whether your thing falls under their remit or not. Right? I’ve decided I’m not going to do that. I’m being very, very honest with you. So I made that call myself. I don’t ask permission.’ *(Director, Interview 17)* |
| ‘But now we have found ourselves in a situation in which the GRC is becoming the guardian and, which is maybe wrong, but it is becoming the guardian perhaps overruling the autonomy of the department.’ *(Coordinator, Interview 3)* |
| ‘I think the GRC is, is not a block at all, certainly in my experiences with them... that it’s very straightforward... if you read the guidance and do it properly, and there’s probably no good reason not to do that, then it’ll sail through.’ *(Technical Officer, Interview 9)* |
| ‘So they were seen as an obstacle, but you know if you then build the process in the work you are doing and then you see actually they’re not an obstacle, they’re a major resource… I wouldn’t think that we would have succeeded without that carriage of process and without that group helping us.’ *(Director, Interview 8)* |
| ‘You know GRC I think has been a good process for [the] WHO and I think that the standards are being set, it remains to be seen how the organisation is going to support GRC, because I think it needs support.’ *(Technical Officer, Interview 20)* |
| ‘I sometimes put it to people who start having this discussion with us, to say we’ve set standards for the way we do business here, if you’re trying to avoid the process and avoid the committee, which of the standards is it that you’re trying not to adhere to. In other words, explain to me, justify why you believe that you can do things in a substandard way, because that’s what this is about.’ *(GRC, Interview 16)* |
