# Supplementary material for: World Health Organization Guideline Development: An Evaluation
Source: PLoS One. 2013 May 31;8(5):e63715. doi: 10.1371/journal.pone.0063715 (PMC3669321; doi:10.1371/journal.pone.0063715)
Supplement: Table S3 — Theme 3: Uncertainties about applying the GRADE methods. (DOCX) [file pone.0063715.s006.docx]

| Theme 3: Uncertainties about applying the GRADE methods |
| --- |
| ‘On the other hand, the actual GRADE process does make it difficult... to address some of the guidance that needs to be provided, and traditionally has been provided by WHO. So the existing process is very useful, say for clinical guidelines, but then when it comes to programmatic guidelines, operational guidelines, there are real, real issues.’ *(Technical Officer, Interview 9)* |
| ‘To do surgery you need the surgical table, but there is no evidence that you don’t need it because there are no randomised control trials on people who are doing surgery without a surgical table, for example. So there are things that are very obvious to everyone but there is no evidence that it shouldn’t be that way.’ *(Coordinator, Interview 19)* |
| ‘It was for the first time that we were actually using GRADE for something in health systems and at the time I remember a lot of criticism from around the house and you can’t really do guidelines in health systems, you can’t have randomised control trials, this is for clinical work, this is not going to work, and so on and so forth but we pushed for it. We tried to follow as much as, as closely as we understood the process and eventually we’d done it and it was really appreciated by the GRC.’ *(Technical Officer, Interview 11)* |
| ‘And the other thing is that WHO has an important role to play in GRADE because GRADE as you know was really developed for clinical questions where there’s clear effect sizes and everyone knows what they’re talking about, and when you start trying to apply that in public health and policy, and fields [like] environmental health where there aren’t any trials and there aren’t really any effect sizes and it’s all observational, it gets difficult. So WHO has to feed that back to GRADE so the methods evolve.’ *(GRC, Interview 12)* |
| ‘I think, some people are not really, don’t really fully understand, have lost sight of the principles; the principles being when we publish guidance, recommendations, whatever we want to call it, it, they should be objective, they should be transparent in the sense that people can see very clearly how we have developed those recommendations and on what evidence we’ve based them, and they should be evidence based.’ *(GRC, Interview 16)* |
