# Supplementary material for: World Health Organization Guideline Development: An Evaluation
Source: PLoS One. 2013 May 31;8(5):e63715. doi: 10.1371/journal.pone.0063715 (PMC3669321; doi:10.1371/journal.pone.0063715)
Supplement: Table S4 — Theme 4: Variable capacity to implement the new standard. (DOCX) [file pone.0063715.s007.docx]

| Theme 4: Variable capacity to implement the new standards |
| --- |
| ’If you have to do the work properly, you need to be trained, and as I said, we don’t have an organisation base of people who are conversant in systematic reviews but you know it seems that any of us, should at least understand what it is.’ *(Director, Interview 8)* |
| ‘But [XX] came in exactly with the skills required to do this, I mean he’s an epidemiologist, been doing systematic reviews, very conversant with the way to do this. Other colleagues were not being exposed to systematic reviews in the past. I found it difficult so they had to go through the learning curve too, including myself.’ *(Director, Interview 8)* |
| ‘I think it was more because we were concerned about the steps and the heaviness of the whole process once again. But then when I started to get feedback from the scientists working with me on, individually and saying, oh, do you know what, we have a briefing and it was extremely useful and I went to the clinic to get advice on how to do this and it was extremely useful. So I think [the GRC] has been contributing as well to change a culture.’ *(Director, Interview 14)* |
| ‘So I think it depends on the technical ability of the people within [the] WHO. If you have a technically weak person, you are completely at the mercy of the experts and the panel, and [that] shouldn’t be the case if we are a technical organisation. And we call ourselves one.’ *(Coordinator, Interview 5)* |
| ‘It [the GRC secretariat] is way too small, yeah. It means we can’t do any training; we can’t do any development work. We can just about keep up with the routine submissions.’ *(GRC, Interview 12)* |
